# Determinants of Desire for Children among HIV-Positive Women in the Afar Region, Ethiopia: Case Control Study

**DOI:** 10.1371/journal.pone.0150566

**Published:** 2016-03-01

**Authors:** Fatimetu Mohammed, Nega Assefa

**Affiliations:** College of Health and Medical Sciences, Haramaya University, Harar, Ethiopia; National Institute of Health, ITALY

## Abstract

**Introduction:**

The desire for a child in Ethiopian society is normal. Among HIV positive women, due to the risk of MTCT, it is imperative to understand factors influencing women’s desire for children. This study aimed at assessing factors associated with desire for children among HIV-positive women in two selected hospitals of Afar Regional State, Ethiopia.

**Methods:**

A facility based case-control study was conducted among 157 cases (with a desire) and 157 controls of HIV positive individuals registered in the selected health facilities. The participants were selected by random sampling technique. Data were collected using face-to-face interview and was analyzed using logistic regression.

**Result:**

Factors found to be independently associated with desire for children were age categories of 20–24 years (OR = 6.22, 1.29–10.87) and 25–29 years (OR = 14.6, 3.05–21.60), being married (OR = 5.51, 2.19–13.54), Afar ethnicity (OR 6.93, 1.19–12.14), having HIV-positive children (OR 0.23, 0.09–0.63), duration on ART more than one year (3.51, 1.68–9.05), CD4 count greater than 350 (OR 4.83, 1.51–7.27) and discussion of reproductive health issues with health providers (OR 0.31, 0.12–0.51).

**Conclusion:**

Women who were young, married, Afar, those who received ART more than one year, and had CD4 count >350 were more likely to have a desire for children.

**Recommendation:**

Health care workers at ART clinic should openly discuss about the reproductive options for the women living with HIV/AIDS.

## Introduction

Globally, half of people living with HIV are women and 76% of all HIV-positive women live in sub-Saharan Africa countries [1]. The infection rate of HIV among pregnant women in Sub-Saharan Africa is alarmingly high. In this part of the world 90% of global new child infections through mother-to-child transmission (MTCT) occur[1]. There is global consensus that the world must strive toward the elimination of new HIV infections among children by 2015 and keep mothers and children living with HIV alive [2]. In Ethiopia, the prevalence of HIV was 1.8% for males and 2.8% for females [3].

High fertility and aspiration to have more children is a normal phenomenon in many developing countries and in Ethiopia too. This is related to the socio-economic situation of people basing on subsistent living. Similar to HIV negative, HIV positive women have the same aspiration to have more children. In many developing countries, where the prevalence of HIV is high and the health system addressing the problems is poorly functioning, the risk of HIV transmission to the baby is inevitable. Despite this risks and challenges, many women living with HIV decides to have children to give purpose for life [4]. But the desire to have children among HIV-infected persons has significant implications for the transmission of HIV to sexual partners and newborns [5].

Before the availability of antiretroviral therapy (ART) in Africa, women infected with HIV not only had reduced fertility, but also reduced fertility aspirations as many women, men, and health providers were opposed childbearing by persons infected with HIV. As access to ART increased, several studies documented a rebound increase in fertility desires. Yet without proper follow-up and medical support the risk of MTCT is high and risks the life of the baby [6–8]

Several studies that have described pregnancy intention rates in different contexts of people living with HIV have reported that the rate of transmission from mother to child ranges from 17% in Uganda to 63% in Nigeria [9, 10]. The situation is nearly similar in Ethiopia, for example in South Wollo, Amhara region it was found to be 18.3% while in Addis Ababa it was 44.7% [11, 12]. It is believed that HIV positive women who had a good knowledge on MTCT tends to consult health personnel at the time of desire to have child or takes necessary precautions not to be pregnant.

Despite the growing importance of fertility issues for HIV-infected women and its implication for HIV related intervention programs, in Ethiopian context, little is known about the fertility desires of women living with HIV [13]. Therefore, the purpose of this study was to assess factors which play a role in the desire for children among women of reproductive age living with HIV.

## Methods

A facility based case-control study was conducted during December 2011 to March 2012 among women of reproductive age group (15–49 years) and attending ART care at Asaita and Dubti hospitals of Afar Region, Ethiopia. Afar is one of the nine regional states in Ethiopia, its capital is Semera, and it is the homeland of the Afar people. The adult HIV prevalence of the region was estimated to be 2.0%, and there were about 17,432 individuals living with HIV/AIDS in need of ART [14]. The total number of female clients who ever started HIV/AIDS care in Asaita and Dubti hospitals was 526 and 1535, respectively.

Ethical clearance for the study was obtained from Institutional Research Ethics Review Committee of College of Health Sciences, Haramaya University and the Afar Regional Health Bureau. Written consent was secured from study participants before conducting the interview. For women, who are married and less than 18 years of age, in addition to the consent form secured from the study participants, their husbands were asked permission to include their wife in the study (sort of assent). But for study participants, who are less than 18 yars of age, were not married, and living independently, as there was no one to sign on behalf of parent or guardian, only consent form was obtained. This condition was communicated to the ethical clearance office, and the study was approved. For illiterate participants the data collectors read the contents of the consent form and asked them to make sure if they have understood the purpose of the study, then they were requested to sign using thumbprint.

Sample size was calculated using the Open Epi epidemiological calculator by considering the following assumptions: 95% CI, 80% power and case-control ratio of 1:1. The final sample size was determined with the proportion of case’s and control’s knowledge of PMTCT which was 27.5% and 14%, respectively [12]. This has yield 157 and 157 for both cases and controls. Study participants were grouped as cases when they had desire to have children and controls when they have no desire to have children. In order to classify study participants as cases and controls, primary assessment was done on all ART clinic attendants one month before the actual data collection was done. Based on this assessment, study participants were grouped as cases if they have desire for additional children and controls were those who reported don’t have desire to have children. Then actual study participants were selected randomly from the list of those who have desire and don’t have desire, respectively.

Data were collected face-to-face by trained nurse interviewer using a pre-tested and structured questionnaire. To ensure quality data, proper training of data collectors, onsite supervision, and proper re-coding of variables.

A cleaned data were entered to the computer using Epi-Info Version 3.5.3. Then it was exported to SPSS Version 16 for analysis. A bivariate analysis was conducted to assess the relationship between the independent variable and the outcome variable; variable with a p-value <0.2 in the crude analysis were put to multiple logistic regression model for final analysis. Odds ratio with a 95% confidence was used to determine the strength of associations. Statistical significance was considered when the p = value is less than 0.05.

## Results

### Socio-demographic characteristics

A total 157 cases and 157 controls were included in the study. The majority of cases and controls were in the age ranges of 25–29 (32.5%) and 15–19 (26.8%), respectively. The two dominant religions (Muslims and Orthodox Christian) constitute 58.6% of cases and 50.3% of controls, respectively. One hundred four (66.2%) of cases and 60(38.2%) controls were married, and 63 (40.1%) of cases and 38 (24.2%) of controls were housewives. Regarding to ethnicity, the majority of cases (45.9%) and controls (25.5%) were Afar. Regarding monthly income of respondents, majority of the cases and controls had a monthly income of 200–500 Ethiopian Birr. From the crude analysis, it is found that married, ethnicity (Afar, Amhara), Muslim, age group (age group of 20–24 and 25–29) were positively and significantly associated with a desire for children (Table 1).

**Table 1:** Socio-demographic characteristics of cases and controls among HIV positive on ART women, Afar, Ethiopia, 2012.

Variables	Cases n (%)	Controls n (%)	Crude OR (95% CI)	P-value
Age				
15–19	21(13.4)	42(20.8)	1.08(0.5–2.55)	0.83
20–24	36(22.9)	14(81.9)	5.60(2.40–13.0)	0.002*
25–29	51(32.5)	23(14.6)	4.83 (2.26–10.28)	0.001*
30–34	32(20.4)	41(26.1)	1.70(0.76–3.80)	0.15*
35–40	17(10.8)	37(23.6)	1.00	
Religion				
Muslim	92(58.6)	53(33.8)	2.89(1.40–5.96)	0.000*
Orthodox	50(31.8)	79(50.3)	1.05(0.50–2.19)	0.410
Protestant	15(9.6)	25(25.9)	1.00	
Marital status				
Married	104(66.2)	60(38.2)	3.17(2.00–5.03)	0.000*
Unmarried	53(33.8)	97(61.8)	1.00	
Ethnicity				
Afar	72(45.9)	40(25.5)	4.25(1.90–9.50)	0.000*
Amhara	39(24.8)	27(17.2)	3.41(1.44–8.05)	0.031*
Oromo	22(14.0)	37(23.6)	1.41(0.66–3.39)	0.49
Tigray	13(8.3)	27(17.2)	1.14(0.43–2.99)	0.79
Guragie	11(7.0)	26(16.6)	1.00	
Educational Status				
Can’t read & write	35(22.3)	22(14.0)	2.18(0.76–6.28)	0.27
Read only	9(5.7)	18(11.5)	3.18(0.21–8.33)	0.42
Read & write only	15(9.6)	19(12.1)	1.9(0.8–4.54)	0.35
Grade 1–4	23(14.6)	21(13.4)	1.45(0.65–3.22	0.31
Grade 5–8	30(19.1)	22(14.0)	1.16(0.54–2.51)	0.69
Grade 9–10	23(14.6)	27(17.2)	1.17(0.86–4.03)	0.77
Grade 11–12	14(9.8)	17(11.5)	1.13(0.84–4.92	0.83
College and above	8(5.1)	11(7.0)	1.00	

* p-values are significant for the crude analysis

### Reproductive history of study participants

Sixty-two (39.5%) of the cases and 41(26.1%) of the controls had no child. Twenty-five (22.1%) of cases and 72(52.9%) of controls had HIV-positive children. Forty-three (36.4%) of cases and 51(37.2%) of controls had ever lost a child.

In bivariate analysis, the odds of desire to have children were significantly higher in women who have no children and whose husband/partner have desire, whereas women who lost child within one year and have HIV-positive child were less likely to have desire for a child (Table 2).

**Table 2:** Reproductive histories of cases and controls included in the study, Afar, Ethiopia, 2012.

Variables	Cases n (%)	Controls n (%)	Crude OR (95%)	p-value
Number of children				
0	62 (39.5)	41 (26.1)	2.59(1.30–3.95)	0.004*
1–2	53(33.8)	51(32.60)	1.57(0.86–2.58)	0.47
≥ 3	42(26.7)	65(41.3)	1	
Child ever lost				
Yes	43(36.4)	51(37.2)	1	
No	75(63.6)	86(62.8)	0.96(0.58–1.61)	0.41
Time of child lost				
< 1 year	9(20.9)	25(49.0)	0.22(0.08–0.65)	0.000*
1–2 years	15(34.9)	14(27.5)	0.68(0.21–1.88)	0.35
> 2 years	19(44.2)	12(23.5)	1	
Have HIV+ child				
Yes	25(22.1)	72(52.9)	0.25(0.14–0.44)*	0.004*
No	88(77.9)	64(47.1)	1	
Partner’s desire				
Yes	84(53.5)	61(38.9)	1.81(1.15–2.83)	0.009*
No	73(46.5)	96(61.1)	1	

* p-values are significant for the crude analysis

### HIV/ART related conditions

Fifty-nine (37.6%) of cases and 82 (52.2%) of controls had less than one year duration of HIV diagnosis. One hundred sixteen (73.9%) cases and 127(80.9%) controls had started ART. Forty-six of cases (29.3%) and 78 (49.7%) controls had a recent CD4 cell count of less than 200. Eighty-two (52.2%) of cases and 114 (72.6%) of controls knew about transmission of HIV from mother to child. Out of them 75(91.4%) of cases and 103(90.3%) of controls knew the availability of medication to prevent mother to child HIV transmission. Sixty-nine (43.9%) of cases and 114 (72.6%) of controls had discussed reproductive health issues with their health providers/counselors. Concerning the overall health conditions after starting ART, 66 (50.4%) of the cases and 39 (26.7%) of the controls had improved health conditions (Table 3).

**Table 3:** HIV/ART related characteristics of cases and controls, Afar, Ethiopia, 2012.

Variable	Cases n (%)	Controls n (%)	Crude OR (95%CI)	p-value
Time of HIV diagnosis				
≤1 year	59(37.6)	82(44.9)	0.55(0.35,0.86)*	0.009*
>1 year	98(62.4)	75(55.1)	1	
Started ART				
Yes	116(73.9)	127(80.9)	0.67(0.38,1.18)	0.29
No	41(26.1)	30(19.1)	1	
Duration on ART				
≤1 year	27(23.3)	74(57.8)	1	
>1 year	89(76.7)	54(42.2)	4.60(2.63,8.03)*	0.001*
Recent CD4 cell count				
<200	46(29.3)	78(49.7)	1	
200–350	36(22.9)	47(29.9)	1.30(0.73,2.28)	0.36
>350	75(47.8)	32(20.4)	3.97(2.39,6.89)	0.000*
Knowledge about PMTCT				
Good	98(64.3)	80(51.0)	1.73(1.10,2.72)*	0.01*
Poor	59(35.7)	77(49.0)	1	
Self reported health condition				
Improved	61(52.6)	33(26.0)	3.12(1.67,5.53)	0.05*
No change	29(25.0)	49(38.6)	1.02(0.52,1.99)	0.27
Deteriorated	26(22.4)	45(35.4)	1	
Discussion on SRH				
Yes	69(43.9)	114(72. %)	0.29(0.18–0.47)	0.003*
No	88(56.1)	43(27.4)	1	

* p-values are significant for the crude analysis

After controlling for possible confounders however, age, marital status (5.51, p-0.00), Afar ethnicity (6.93, p-0.03), having HIV-positive children (0.23 p-<0.05), duration on ART (3.51, p-0.003), CD4count (4.83, p-0.008), and discussion of reproductive health issues (0.31, p-0.01) were significantly associated with having fertility desire compared to their counter parts. Women who were young, married, Afar, spent greater than one year on ART and had CD4 count > 350 were more likely to have desire for children. However, women who had HIV-positive child and had discussed about RH issues with their health providers were less likely desire to have children. PMTCT knowledge though in the crude analysis shows a significant association, it turns out to be insignificant in the final analysis. (Table 4)

**Table 4:** Factors independently associated with desire for children among cases and controls of HIV positive women on ART Afar region, Ethiopia, 2012.

Variables	Crude OR (95%CI)	Adjusted OR (95%CI)
Age		
15–19	1.08(0.5–2.55)	0.70(0.16–3.10)
20–24	5.60(2.40–13.0)*	6.22(1.29–10.87)**
25–29	4.83 (2.26–10.28)*	14.6(3.05–21.60)**
30–34	1.70(0.76–3.80	2.05(0.53–4.79)
35–40	1.00	1.00
Religion		
Muslim	2.89(1.40–5.96)*	1.47(0.89–4.51)
Orthodox	1.05(0.50–2.19)	0.71(0.26–1.86)
Protestant	1.00	1.00
Marital status		
Married	3.17(2.01–5.03)*	5.51(2.19–13.54)**
Unmarried	1.00	1.00
Ethnicity		
Afar	4.25(1.90–9.50)*	6.93(1.19–12.14)**
Amhara	3.14(1.44–8.05)*	2.63(0.43–4.10)
Oromo	1.41(0.66–3.39)	2.10(0.32–5.50)
Tigray	1.14(0.43–2.99)	0.31(0.16–2.84)
Guragie	1.00	1.00
Number of children		
0	3.59(1.32–5.06)*	1.7(0.72–4.01)
1–2	1.77(0.97–3.24)	0.81(0.76–3.62)
≥ 3	1.00	1.00
Time of child loss		
< 1 year	0.22(0.08–0.65)*	0.14(0.01–1.04)
1–2 years	0.68(0.21–1.88)	1.03(0.16–6.44)
> 2 years	1.00	1.00
Partner’s desire		
Yes	1.81(1.15–2.83)*	1.04(0.68–1.94)
No	1.00	1.00
Having HIV+ Children		
Yes	0.25(0.14–0.44)*	0.23(0.09–0.63)**
No	1.00	1.00
Time of HIV diagnosis		
≤1 year	0.55 (0.35–0.86)*	0.49(0.11–2.21)
>1 year	1.00	1.00
Duration on ART		
≤ 1 year	1.00	1.00
> 1 year	4.60(2.63–8.03)*	3.51(1.68–9.05)**
Recent CD4 cell count		
<200	1.00	1.00
200–350	1.30(0.73–2.28)	0.39(0.13–1.17)
>350	3.97(2.39–6.89)*	4.83(1.51–7.27)**
Self reported Health Condition		
Improved	3.12(1.67–5.53)*	1.74(0.91–6.02)
No change	1.02(0.52–1.99)	0.59(0.31–2.40)
Deteriorated	1.00	1.00
Knowledge about PMTCT		
Good	1.73(1.10–2.72)*	1.19(0.38–1.24)
Poor	1.00	1.00
Discussion on SRH issues		
Yes	0.29(0.18–0.47)*	0.31(0.12–0.51)**
No	1.00	1.00

* Significant in Bivariate analysis

** Significant in Multiple logistic regression analysis

## Discussion

Understanding the desire for children in women living with HIV/AIDS is important to reduce MTCT especially with the introduction of ART which changes views of childbearing despite having the disease [15].

The age of the women was significantly associated with desire for children. Women whose age ranges fell into 20–29 had higher desire for children. It is quite normal to have a desire to have a child during this period. It is the pick of reproduction. In many societies, women are active in producing their off springs during this period. Other studies in Addis Ababa have also produced similar findings [16].

Being an Afar in ethnicity was positively associated with a desire for children. This might be from the high prevalence of polygamy and high desire to have a child among Afar community and woman may have felt competitive with the other wives by having more children. In such society children are considered assets to assist with the traditional cattle raring activities among families. In such societies, women who have more children are respected in the society [17, 18].

In bivariate analysis, number of children and lost children were significantly associated with the desire for child, but they lost association in the adjusted analysis. This finding is Contrary to the result in Addis Ababa study and studies done in Uganda and Brazil which have showed a significant association with the desire for child [16, 19, 20]. This difference might come from differences in research methods.

Women who have HIV-positive child showed less desire for additional child. This might be due to fear of having an additional HIV-positive child or fear of losing a child from AIDS related disease. No similar or opposing results were found from other researches; hence it is potential area for further research.

Duration on ART had a significant association with desire for a child; women who spent more than one year on ART had a greater desire for children than their counterparts. This might be due to the dramatic reversal of disease progress and improvement in quality of life that had created hope and reduces the fatalistic attitude of the women to HIV/AIDS. In addition, those who stayed more years on ART might think that, the longer they are on ART is the less likely to have HIV-positive children. In studies conducted in South Africa and Uganda, being on ART or pre-ART was associated with the desire for children [21, 22].

In this study, women whose recent CD4 cell count is greater than 350 cells/μl had a greater desire for a child. This might be from feeling of wellbeing followed with high immunity status and fewer opportunistic infections. This finding is consistent with the study conducted in South Africa and India [21]. A patient with CD4 cell count below 200cells/μl are at increased risk of developing life threatening opportunistic diseases [23].

Discussions with health providers on reproductive health like sexuality and childbearing issues were associated with a desire for child. Women who discussed this issue with the health professionals were less likely to have a desire for more children. This might be due to their understanding of child bearing within the context of HIV positivity.

## Conclusions and Recommendations

A desire to have additional child is significantly associated with age, ethnicity, marital status, duration on ART, and CD4 count. Whereas having HIV positive child and discussion about reproductive health issues with health provides were negatively associated with a desire to have a child.

Health providers who work in ART clinic should openly discuss sexual and reproductive health issues with their clients. They should provide adequate information about reproductive options for women living with HIV/AIDS to assist them in making informed reproductive decisions.

NGOs who work on HIV/AIDS care and support should focus on promoting and strengthening reproductive health services provided in the HIV/AIDS care units. Also case managers should be fully aware of the culture and norms of Afar society to address these groups properly and in a sensitive manner when communicating sexuality and childbearing issues.
